# Realización de pruebas innecesarias en los ensayos clínicos: hCG como ejemplo

**DOI:** 10.1515/almed-2019-0044

**Published:** 2020-04-07

**Authors:** Estibaliz Alegre, Miguel F. Sanmamed, Teresa Sendino, Jose Luis Perez-Gracia

**Affiliations:** Biochemistry Laboratory, Clínica Universidad de Navarra, Avenida Pío XII 36, 31008, Pamplona, Spain; Departamento de Oncología, Clínica Universidad de Navarra, Pamplona, Spain; Laboratorio de Bioquímica, Clinica Universidad de Navarra, Pamplona, Spain

**Keywords:** ensayo clínico, gonadotropina coriónica (hCG), solicitud de pruebas

Estimado Editor,

Entre los requisitos para ser incluido en un ensayo clínico, los pacientes tienen que someterse a una serie de análisis de laboratorio. Estos análisis son obligatorios y suelen solicitarse de manera sistemática para todos los pacientes, independientemente de su situación clínica. Estos análisis son condición *sine qua non* para que un paciente pueda ser incluido en un ensayo, pero su petición indiscriminada ajena al contexto clínico puede tener consecuencias indeseadas para los pacientes. Además de las pruebas de función renal, la gonadotropina coriónica (hCG) es una de las pruebas que se suelen solicitar con mayor frecuencia, para evitar que las mujeres embarazadas entren o continúen en un ensayo clínico.

La hCG es la hormona utilizada habitualmente en las pruebas de embarazo por su alta especifidad. Sin embargo, cuando esta prueba se solicita sin tener en cuenta el contexto clínico, puede dar resultados engañosos y dificultar la inclusión de una paciente en un ensayo clínico.

Un ejemplo de ello es cuando se realiza la prueba de hCG a mujeres posmenopáusicas, que pueden presentar valores de hCG en plasma por encima del punto de corte establecido para la detección del embarazo. En nuestro laboratorio hemos observado dicho incremento en el 8.3% de las mujeres mayores de 45 años que se sometieron a una prueba de hCG antes de ser incluidas en un ensayo clínico. Estos valores ligeramente elevados (<25 UI/L) se pueden atribuir a la posible producción de hCG por la hipófisis. Por ello, podrían establecerse puntos de corte más elevados para las mujeres postmenopáusicas [1]. En cualquier caso, sería preciso analizar los niveles de hormona estimulante del folículo (FSH) y hormona luteinizante (LH) para confirmar la situación de postmenopausia [2].

El aumento de hCG también puede ser debido a una producción ectópica por diferentes tipos de tumores. En este caso, los niveles de hCG serán superiores a los observados en las mujeres postmenopáusicas (por encima de los 100 UI/L). Como en el caso anterior, se deberían realizar también análisis adicionales para evitar la exclusión de una paciente de un ensayo clínico como consecuencia de un falso positivo en el test de embarazo. Esta situación puede ser aún más confusa, ya que los análisis de LH and FSH pueden no ser de utilidad si la mujer no es postmenopáusica. En esos casos habría que comprobar si los niveles elevados de hCG son debidos en realidad a niveles altos de la subunidad beta de hCG libre, ya que algunos de los kits disponibles en el mercado para la cuantificación de hCG no solo detectan el heterodímero completo de hCG, sino también la subunidad beta libre. Esta verificación es de utilidad, ya que se ha descrito la producción ectópica de la subunidad beta libre en casos de cáncer epitelial [3], [4].

En ambas situaciones, puede que no se excluya finalmente a las pacientes del ensayo clínico a pesar de presentar niveles elevados de hCG, pero sí se provocaría una demora en el proceso de inclusión, ya que se requerirían análisis adicionales para identificar la causa de dicha elevación. Esta demora se podría haber evitado si no se hubiese solicitado la realización de estos análisis a mujeres en las que una gestación no es posible, principalmente debido a su edad.

Algunos autores ya han cuestionado que se utilicen los resultados de los análisis de hCG como criterio de exclusión [5]. De hecho, la mayoría de los protocolos actuales no suelen indicar la necesidad de realizar un análisis de hCG en sí mismo, sino que es el clínico el que debe descartar la posibilidad de un embarazo. La posibilidad de embarazo no solo se puede evaluar midiendo hCG, sino teniendo en cuenta también la edad del paciente y/u otras circunstancia, como haberse sometido a una histerectomía. Por consiguiente, la solicitud indiscriminada de hCG por parte de las Unidades de Ensayos Clínicos no está siempre justificada.

En conclusión, se debe fomentar que los laboratorios adopten una actitud proactiva y comenten con el médico solicitante la conveniencia de realizar los análisis de hCG en algunas pacientes. El hecho de que se trate de un ensayo clínico no justifica que se soliciten algunas pruebas cuyos resultados pueden ser engañosos o incluso absurdos en algunos contextos clínicos. Una comunicación fluida entre analistas y los clínicos podría reducir la realización de análisis innecesarios, así como prevenir las posibles inconveniencias que estos puedan causar.
